# Genomic and Epidemiologic Insights into Ongoing Measles Outbreak, Israel, 2025–2026

**DOI:** 10.3201/eid3209.260625

**Published:** 2026-09

**Authors:** Efrat Bucris, Neta S. Zuckerman, Tal Levin, Yara Kanaaneh, Oran Erster, Victoria Indenbaum, Keren Friedman, Danit Sofer, Eli Schwartz, Elena Chernin, Or Kriger, Rana Shibli, Sharon Elroi Prais, Zohar Mor, Nitza Abramson, Michal Savion, Naama Nuss, Ady Cohen Golan, Eva Avramovich, Dror Yakir, Liora Guy David, Efrat Rorman, National Measles Working Group,, Yaniv Lustig

**Affiliations:** Central Virology Laboratory, Public Health Services, Ministry of Health and Sheba Medical Center, Ramat-Gan, Israel (E. Bucris, N.S. Zuckerman, T. Levin, Y. Kanaaneh, O. Erster, V. Indenbaum, K. Friedman, D. Sofer, Y. Lustig); Gray Faculty of Medicine, Tel-Aviv University, Tel-Aviv, Israel (N.S. Zuckerman, Y. Lustig); The Center for Geographic Medicine and Tropical Diseases, The Chaim Sheba Medical Center, Tel Hashomer, Israel (E. Schwartz); Ministry of Health Division of Epidemiology, Jerusalem, Israel (E. Chernin, O. Kriger, R. Shibli, S.E. Prais); Health Intelligence, Ministry of Health, Jerusalem (S.E. Prais, D. Yakir, L.G. David); Program for Public Health, Ashkelon Academic College, Ashkelon, Israel (Z. Mor); Jerusalem Department of Health, Ministry of Health, Jerusalem (Z. Mor, N. Abramson); Tel Aviv Department of Health, Ministry of Health, Tel-Aviv (M. Savion, N. Nuss); Central District Health Bureau, Ramla, Israel (A.C. Golan, E. Avramovich); Ministry of Health Department of Laboratories, Jerusalem (E. Rorman)

**Keywords:** measles, viruses, vaccine-preventable diseases, outbreak, molecular diagnosis, phylogenetic analysis, Israel

## Abstract

An ongoing measles outbreak in Israel, involving ≈3,200 cases and 16 deaths, threatens the country’s measles elimination status and reflects declining vaccination rates observed globally and within Israel. Epidemiologic investigations supported by sequencing suggest that a single importation triggered the outbreak, underscoring the critical role of rapid genomic surveillance in outbreak control.

Measles is a highly contagious, vaccine-preventable disease; clinical manifestations typically include high fever, maculopapular rash, cough, and conjunctivitis. Severe complications, including pneumonia and encephalitis, can occur in up to 30% of cases, and the case-fatality rate is ≈1/1,000 cases (*1*). Despite an effective vaccine (measles, mumps, and rubella [MMR]), measles remains a major global health concern; in 2025, Romania, Vietnam, Morocco, and the United States reported outbreaks (*2*–*4*). 

In Israel, MMR vaccine coverage has declined in recent years to 83% receiving the first dose at <25 months of age and 87% a second dose at <8 years of age. Corresponding rates are 83% and 85% in Jerusalem and 89% and 93% in the Central District; coverage is lower in Ultra-Orthodox communities (Israel Ministry of Health, unpub. data). Israel achieved measles elimination in 2015, but outbreaks continue, including >4,000 cases in 2018–2019 and smaller outbreaks during 2023–2024 (*5**–**7*). We report on an ongoing measles outbreak that began in Israel in April 2025.

## The Outbreak

During April 7, 2025–February 28, 2026, a total of 3,203 measles cases, including 16 deaths, were reported across Israel, the largest outbreak since 2018–2019 (*8*). Epidemiologic investigations found 13 case-patients had a history of travel abroad shortly before illness onset (Table). Among reported case-patients, mean age was 5.58 years (SD 9.35 years; range 1 month–73 years); 82.7% were unvaccinated, 7.1% were partially vaccinated, 2.2% were fully vaccinated, and 8.5% had unknown vaccination status.

**Table:** Patient data from investigation of genomic and epidemiologic insights into ongoing measles outbreak, Israel, 2025–2026*

Patient no.	Case ID	Symptom onset date	Date of return	Country of infection	Epidemiologic data	Sequence available	Genotype
1	597/25	2025 Apr 5	2025 Mar 30	Serbia	Transmission chain of 2 cases	Y	D8
2	681/25	2025 Apr 10	2025 Apr 4†	Belgium	Index case	N	NA
3	651/25	2025 Apr 16	2025 Apr 3	Vietnam	Single imported case	Y	B3
4	835/25	2025 Apr 30	2025 Apr 21	Russia	Transmission chain of 2 cases	Y	B3
5	1813/25	2025 Jun 15	2025 Jun 11	Belgium	NA	Y	B3
6	1929/25	2025 Jul 10	2025 Jul 5	Thailand	Transmission chain of 2 cases	Y	D8
7	3067/25	2025 Aug 26	2025 Aug 22	Poland	Virus likely acquired in Israel	Y	B3
8	3425/25	2025 Oct 2	2025 Sep 30	Ukraine	NA	N	NA
9	4319/25	2025 Oct 3	2025 Oct 10	Poland	Single import-associated case	N	NA
10	4130/25	2025 Oct 31	NA	Ethiopia	Virus likely acquired in Israel	Y	B3
11	5745/25	2025 Nov 5	2025 Oct 22	India	Single import-associated case	N	NA
12	4988/25	2025 Nov 27	2025 Nov 30	Germany	Virus likely acquired in Israel	Y	B3
13	833/26	2026 Jan 11	2026 Jan 8	Greece	NA	N	NA

*ID, identification; NA, not available. †Date of return is estimated; a sample from a close contact was sequenced and determined as B3 genotype.

As part of routine surveillance, 132 measles virus–positive samples collected during April 2025–February 2026 were selected for sequencing (Appendix). Samples were sequenced on the basis of the following criteria: all available imported cases, all travel-associated cases, representative samples from newly affected areas, and randomized samples collected in established outbreak locations every 2 weeks throughout the outbreak. Samples were excluded from sequencing when epidemiologic data showed that transmission occurred in established measles-positive locations (i.e., same street or household) (Appendix). Complete measles virus genomes were sequenced (*9*) (Appendix) and used for phylogenetic analysis to support transmission chain reconstruction. The N450 region of the measles viral genome confirmed the sequences as genotype B3, matching the reference MVs/Quetta.PAK/44.20 strain from Pakistan.

**Figure 1:**
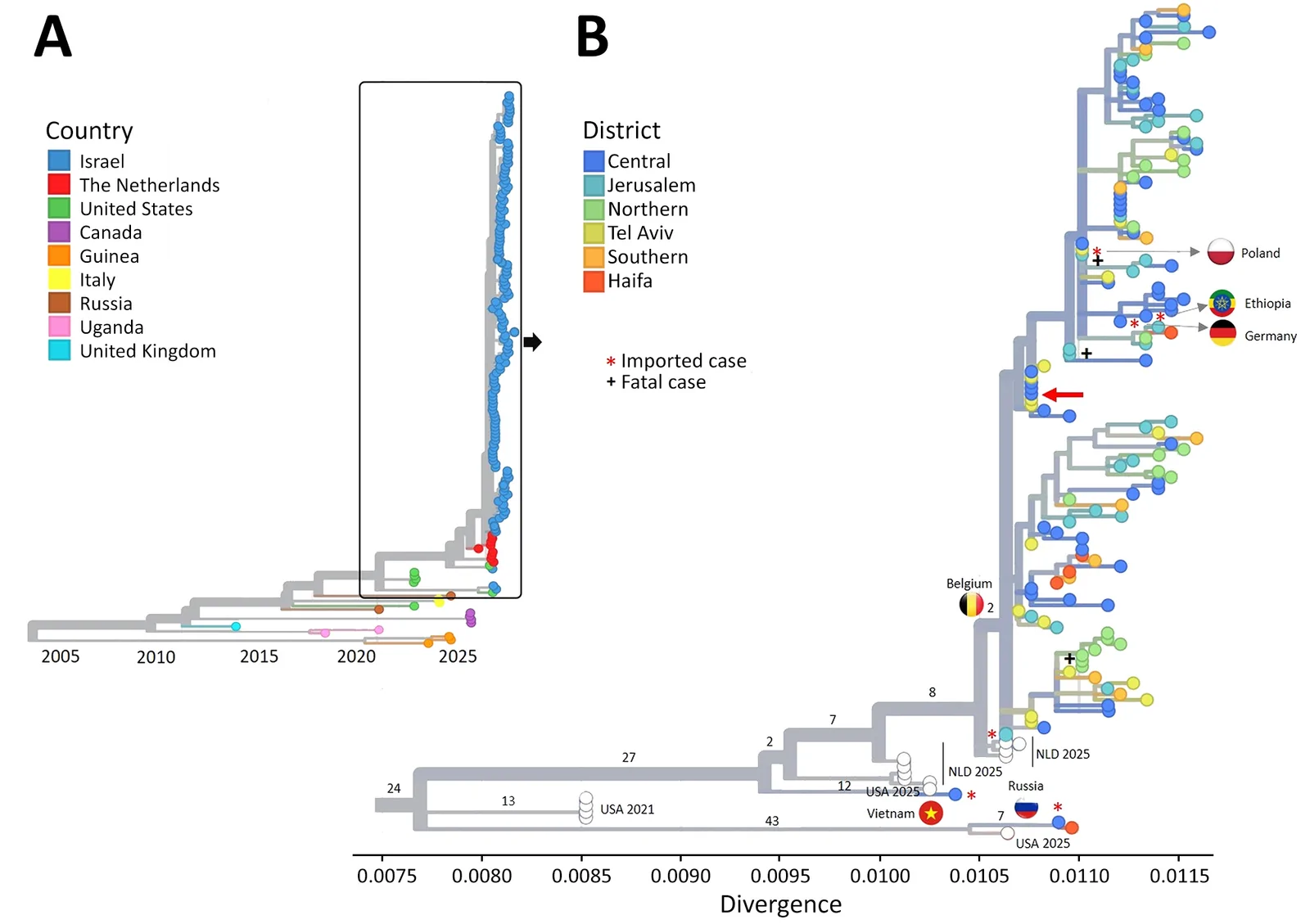
Phylogenetic analysis from investigation of genomic and epidemiologic insights into ongoing measles outbreak, Israel, 2025–2026. Phylogenetic tree including 132 sequences from Israel collected during April 2025–February 2026 and 29 global sequences from 2020–2025 available in the National Center for Biotechnology Information (Appendix) Tree was constructed using the Nextrain Augur pipeline under the generalized time reversible substitution model, and visualized with Auspice (*10*). A) Time-resolved phylogenetic tree including Israel and global sequences. B) Divergence tree highlighting the Israel clusters and indicating the district of origin for each sample; branch lengths and the x-axis represent genetic divergence, whereas numbers along branches indicate inferred nucleotide substitutions relative to the parental node. Asterisks (*) denote cases with documented travel around the time of measles identification; country flags indicate the reported travel destination when known. Plus signs (+) denote fatal cases in children. Red arrow denotes the epidemiologically linked case related to the initial importation from Belgium.

**Figure 2:**
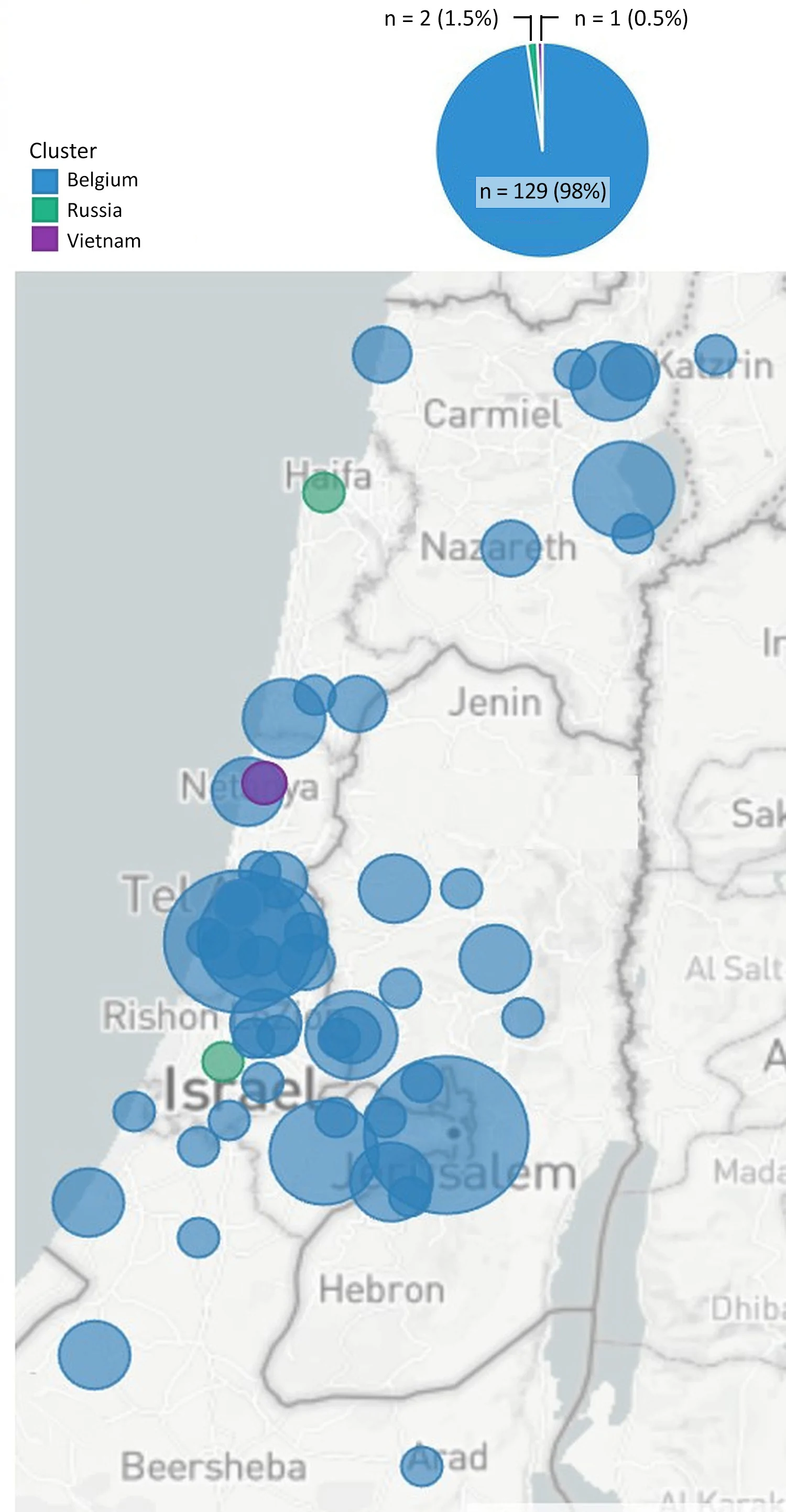
Geographic distribution of sequenced measles cases and importation clusters from genomic and epidemiologic investigation into ongoing measles outbreak, Israel, 2025–2026. Map displays the geographic distribution of sequenced cases by city. Colors denote the 3 major identified importation clusters, and the accompanying pie chart represents the percentage of sequenced samples belonging to each outbreak cluster; number of samples sequenced out of 132 total samples are shown.

Phylogenetic analysis showed the Israel sequences clustered most closely with sequences from the Netherlands and the United States collected in 2025 (Figure 1); however, because up-to-date global sequence data in public repositories are limited, intermediate diversity could be missing. The Israel sequences showed 3 likely clusters within the dominant B3 lineage, each derived from a separate documented importation: Belgium, Vietnam, and Russia. We found no spread of D8 lineage sequences imported to Israel. The Russia cluster likely originated from a documented importation from Russia (patient 4; Rehovot, May 2025) and is linked to an additional sample collected in Haifa in June 2025. That cluster is most closely related to a global sequence from California, USA (2025), differing by 7 nucleotides. Transmission in the cluster was limited to only 2 cases because close contacts were fully vaccinated and that location has high overall vaccine coverage. The Vietnam cluster, separated from the nearest ancestor by 12 nt, likely originated from a documented importation from Vietnam (patient 3; Beit-Itzhak, April 2025) and is not linked to additional Israel cases in the phylogeny. No further transmission was documented in the cluster, consistent with sequencing results and similar to the Russia cluster. The main and largest cluster (n = 129) was traced through epidemiologic investigations to a documented importation from Belgium (patient 2; Modi’in Illit, April 2025). That case was diagnosed retrospectively through contact tracing of infected close family members. Although the imported case was confirmed serologically (positive IgM), the available specimen contained insufficient viral RNA, precluding sequencing. However, sequencing was available for an epidemiologically linked close contact of the imported case (Bnei-Brak, April 2025; Figure 1, red arrow), located within the main cluster; epidemiologic data indicated subsequent spread to additional cities in Central District, Jerusalem, and later North District. That cluster also includes an additional sequenced importation from Belgium (case 5; Jerusalem, June 2025), unrelated epidemiologically to the other Belgium cases, located on the ancestral branch. The main cluster is most closely related to global sequences from the Netherlands collected in 2025, differing by 2 mutations. That strain has subsequently spread in Israel and is still circulating, accumulating mutations and giving rise to several sublineages. Most samples in that cluster were from locations with low vaccine coverage and within Ultra-Orthodox communities (Figure 2; Appendix Table 3). 

The sequences obtained from deceased children were located within different sublineages in the main outbreak cluster (Figure 1). The first case (Jerusalem, July 2025) was identical to 2 sequences detected elsewhere (Tel Aviv, September 2025; Emmanuel, August 2025). The second case (Jerusalem, July 2025) was identical to another from Jerusalem (June 2025). The third case (Poriya, November 2025) was identical to 2 cases from the North District (Nahariya, November 2025). The identical genomes suggest close epidemiologic linkage and, when detected in different districts, indicate transmission across multiple regions in Israel. That finding suggests the virus in fatal cases was typical of the outbreak strain. Three additional case-patients with documented travel abroad (patient 7, Poland; patient 10, Ethiopia; patient 12, Germany) near the time of measles detection had sequences within the main cluster, suggesting that infection was more likely acquired in Israel, not abroad.

**Figure 3:**
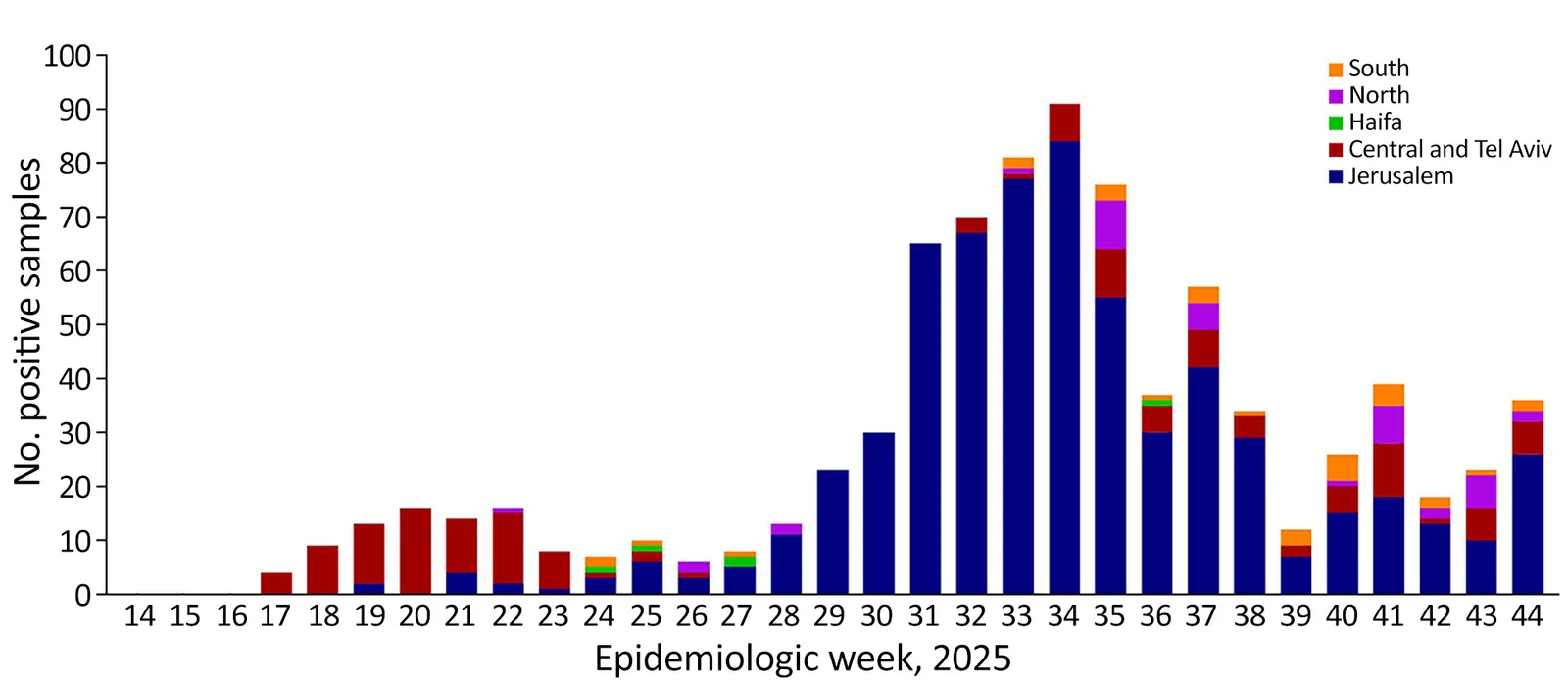
Positivity rates by epidemiologic week during April 1–October 31, 2025, from genomic and epidemiologic investigation into ongoing measles outbreak, Israel, 2025–2026. Weekly number of measles cases by district was confirmed by quantitative reverse transcription PCR.

To investigate positivity dynamics from an epidemiologic perspective, we evaluated the weekly ratios of positive and negative samples collected during April–October 2025 (epidemiologic weeks 14–44). When the outbreak began, the positivity rate was low (10%–20%), likely reflecting heightened awareness and broad testing of suspected cases with low pretest probability for measles. As transmission became established in specific districts during weeks 28–38, positivity increased to ≈60%, indicating outbreak persistence and targeted sampling (Appendix). During weeks 39–44, as the outbreak expanded to additional districts, the overall ratio decreased (Figure 3); we observed a similar trend through February 2026 (data not shown).

## Conclusions

The ongoing measles outbreak in Israel threatens the country’s elimination status, defined by the World Health Organization as absence of endemic transmission for >12 months (https://iris.who.int/server/api/core/bitstreams/b0ee4708-ba01-4b40-9003-f6f1aba317da/content). This outbreak highlights the profound risks associated with declining vaccination coverage. Genomic sequencing of targeted measles samples indicated that a single importation event was most likely the primary driver of the outbreak. Phylogenetic analysis showed 3 validated distinct importation events from Russia, Vietnam, and Belgium; outcomes differed drastically based on local immunity. The main Belgium-linked cluster strain, detected in April 2025, likely sparked an extensive national outbreak. The second documented importation case from Belgium might have contributed to the main transmission cluster. The epidemiologic investigation indicated that this case-patient had many contacts and resided in an area experiencing a substantial outbreak. However, the case was detected 2 months after the Belgium index case, so it likely had little impact on outbreak dynamics. Furthermore, its shared mutations with the April 2025 Belgium case suggest a common source.

Additional undetected single cases might exist because of underreporting. However, our strict criteria and direct contact to Ministry of Health epidemiologic data enabled systematic assessment of each focal point, so underreporting is unlikely to have substantially affected the main phylogenetic findings. Positivity rates rose from 10%–20% during early broad testing to 60% during weeks 28–38, as diagnostics shifted to targeted surveillance during outbreak spread.

In summary, we integrated epidemiologic investigations with molecular surveillance, demonstrating that a single case can spark extensive measles transmission. Our findings highlight the critical role of molecular surveillance in measles outbreak management.

Appendix Additional information for investigation of genomic and epidemiologic insights into ongoing measles outbreak, Israel, 2025–2026.
