# Supplementary material for: Genomic and Epidemiologic Insights into Ongoing Measles Outbreak, Israel, 2025–2026
Source: Emerg Infect Dis. 2026 Sep;32(9):1478–82. doi: 10.3201/eid3209.260625 (PMC13555727; doi:10.3201/eid3209.260625)
Supplement: Supplementary file 1 [file 26-0625-Techapp-s1.pdf]

Article DOI: https://doi.org/10.3201/eid3209.260625

EID cannot ensure accessibility for supplementary materials supplied by authors. Readers who have difficulty accessing supplementary content should contact the authors for assistance.

Genomic and Epidemiologic Insights into Ongoing Measles Outbreak, Israel, 2025–2026

Appendix

Real time PCR

Real-time PCR was performed using an in-house multiplex assay targeting measles virus, vaccine-strain Measles, rubella virus, and RNase P, or the Quanty Measles commercial kit (Clonit, Italy), according to the manufacturer's instructions. All assays were run on Bio-Rad CFX96 instruments.

Extraction, library preparation, sequencing and analysis

Total nucleic acids from clinical samples positive for measles virus were extracted using magLEAD 12gC (PSS, Precision System Science, Matsudo, Japan). Reverse transcription and amplification of the complete measles virus genome were carried out in a single one-step RT-PCR reaction using a previously published set of primers designed to amplify the full viral genome (*1*). To enhance whole genome coverage, the primer mix was supplemented with additional published primers targeting the MF-NCR region (*2*), as well as primers designed to improve coverage of the 3′ genomic region (*3*). PCR products were subsequently used for library preparation with the DNAprep kit (Illumina, CA, USA) according to the manufacturer's instructions on a Hamilton STAR liquid handling robot. Sequencing was performed on the Illumina MiSeq i100 pro platform (Illumina, CA, USA).

Selected samples for sequencing

A suspected measles case is defined according to WHO criteria as a patient presenting with fever and generalized maculopapular (non-vesicular) rash, or any patient in whom a health

care worker suspected measles infection. Of 3203 confirmed measles cases between April 2025 and February 2026, 80% were laboratory confirmed. A total of 1599 samples were available at the national measles center, Central Virology Laboratory, where this study was conducted. Of these, 1090/1599 were suitable for sequencing, defined as samples with Cq =< 25 to ensure full sequence coverage. A total of 132 positive specimens were selected for sequencing as part of routine surveillance, as described in the main text, and are stratified by district and summarized in Appendix Table 2.

Bioinformatic analysis

Fastq files underwent quality control assessment using fastQC and MultiQC (*4*) and low-quality sequences were filtered using trimmomatic (H. Li, unpub. data, http://arxiv.org/abs/1303.3997). Reads were then aligned to an annotated MeV genotype B3 reference genome (KT732215.1) using BWA-mem (*5*). The resulting alignments were processed with the SAMtools suite for sorting, indexing, and additional quality control (*6*). Consensus genome sequences were generated using iVar (*7*), with positions supported by fewer than ten reads masked as 'N'.

For the phylogenetic analysis, the Nextstrain Augur pipeline (*7*) was used as follows. Sequences were aligned to the B3 reference genome using MAFFT (*8*), followed by construction of a time-resolved phylogenetic tree using IQ-TREE and TreeTime (*9*,*10*), employing the GTR substitution model. Final visualization was performed with Auspice.

Appendix References

1. Mulders MN. Annex C16: CDC Protocol for amplification and sequencing of measles virus MF-NCR from patient samples [cited 2022 Jul 12]. https://www.technet-21.org/en/resources/document/annex-c16-cdc-protocol-for-amplification-and-sequencing-of-measles-virus-mf-ncr-from-patient-samples.

2. Indenbaum V, Bucris E, Friedman K, Kushnir T, Eliyahu H, Azar R, et al. Measles Sequencing: Lessons Learned from a Large-Scale Outbreak. Viruses. 2025;17:913. PubMed https://doi.org/10.3390/v17070913

3. Ewels P, Magnusson M, Lundin S, Käller M. MultiQC: summarize analysis results for multiple tools and samples in a single report. Bioinformatics. 2016;32:3047–8. PubMed https://doi.org/10.1093/bioinformatics/btw354

4. Bolger AM, Lohse M, Usadel B. Trimmomatic: a flexible trimmer for Illumina sequence data. Bioinformatics. 2014;30:2114–20. PubMed https://doi.org/10.1093/bioinformatics/btu170

5. Li H, Handsaker B, Wysoker A, Fennell T, Ruan J, Homer N, et al.; 1000 Genome Project Data Processing Subgroup. The Sequence Alignment/Map format and SAMtools. Bioinformatics. 2009;25:2078–9. PubMed https://doi.org/10.1093/bioinformatics/btp352

6. Grubaugh ND, Gangavarapu K, Quick J, Matteson NL, De Jesus JG, Main BJ, et al. An amplicon-based sequencing framework for accurately measuring intrahost virus diversity using PrimalSeq and iVar. Genome Biol. 2019;20:8. PubMed https://doi.org/10.1186/s13059-018-1618-7

7. Hadfield J, Megill C, Bell SM, Huddleston J, Potter B, Callender C, et al. Nextstrain: real-time tracking of pathogen evolution. Bioinformatics. 2018;34:4121–3. PubMed https://doi.org/10.1093/bioinformatics/bty407

8. Katoh K, Misawa K, Kuma K, Miyata T. MAFFT: a novel method for rapid multiple sequence alignment based on fast Fourier transform. Nucleic Acids Res. 2002;30:3059–66. PubMed https://doi.org/10.1093/nar/gkf436

9. Sagulenko P, Puller V, Neher RA. TreeTime: Maximum-likelihood phylodynamic analysis. Virus Evol. 2018;4:vex042. PubMed https://doi.org/10.1093/ve/vex042

10. Nguyen L-T, Schmidt HA, von Haeseler A, Minh BQ. IQ-TREE: a fast and effective stochastic algorithm for estimating maximum-likelihood phylogenies. Mol Biol Evol. 2015;32:268–74. PubMed https://doi.org/10.1093/molbev/msu300

Appendix Table 1. Selected samples for sequencing by district

District	Total Positive Samples	Total suitable samples for sequencing (<=25 cq)	# Sequenced samples(% of total suitable samples)	Remarks
Jerusalem	924	756	27 (4%)	Due to known epidemiological data indicating extensive transmission within specific households, streets and communities there was no need for extensive sequencing per the sequencing criteria.
North	235	87	21 (26%)	
Central	196	113	53 (47%)	Multiple areas without significant epidemiological data resulted in high need for sequencing.
Tel Aviv	134	82	16 (20%)	
South	62	35	10 (29%)	
Haifa	48	17	5 (29%)	
Total	1599	1090	132	

Appendix Table 2. Sequencing coverage for sequenced samples by district

Sequence ID	Sequencing coverage (%)	District
1065-25	93	Central
1095-25	96	Central
1096-25	93	Central
1211-25	93	Central
1334-25	93	Jerusalem
1394-25	98	Central
1427-25	93	Central
1440-25	93	Haifa
1511-25	100	Central
1530-25	94	Southern
1534-25	93	Southern
1545-25	86	Haifa
1568-25	100	Jerusalem
1575-25	90	Central
1576-25	93	Central
1578-25	93	Tel Aviv
1582-25	94	Jerusalem
1583-25	93	Tel Aviv
1691-25	93	Haifa
1711-25	93	Jerusalem
1952-25	100	Jerusalem
2145-25	81	Tel Aviv
2197-25	95	Jerusalem
2231-25	98	Jerusalem
2298-25	92	Northern
2324-25	92	Central
2374-25	78	Tel Aviv
2413-25	93	Central
2435-25	82	Central
2490-25	95	Central
2543-25	90	Southern
2577-25	65	Southern
2579-25	86	Northern
2592-25	82	Central
2629-25	84	Central
2705-25	82	Central
2744-25	100	Southern
2750-25	92	Jerusalem
2754-25	88	Jerusalem
2755-25	100	Central
2839-25	80	Jerusalem
2991-25	100	Northern
3005-25	100	Northern
3019-25	85	Tel Aviv
3039-25	100	Central
3047-25	95	Southern
3152-25	97	Jerusalem
3256-25	84	Central

Sequence ID	Sequencing coverage (%)	District
3337-25	94	Central
3350-25	100	Tel Aviv
3425-25	95	Northern
3449-25	100	Northern
3506-25	100	Jerusalem
3554-25	100	Central
3582-25	100	Southern
3610-25	100	Central
3809-25	100	Jerusalem
4062-25	100	Jerusalem
651-25	93	Central
655-25	96	Tel Aviv
657-25	97	Tel Aviv
662-25	93	Tel Aviv
663-25	93	Central
672-25	99	Central
687-25	99	Central
703-25	95	Central
735-25	93	Tel Aviv
745-25	98	Tel Aviv
747-25	94	Tel Aviv
784-25	99	Central
789-25	93	Jerusalem
835-25	93	Central
890-25	93	Haifa
916-25	97	Central
3988-25	99	Central
3980-25	98	Southern
4072-25	97	Southern
4070-25	99	Northern
4133-25	99	Central
4130-25	99	Central
4249-25	96	Central
4448-25	100	Central
4495-25	97	Northern
4489-25	100	Northern
4378-25	100	Jerusalem
4382-25	100	Northern
4486-25	100	Northern
4660-25	100	Jerusalem
4749-25	100	Northern
891854-25	100	Northern
4750-25	96	Tel Aviv
4753-25	99	Northern
4813-25	96	Southern
4863-25	96	Tel Aviv
4947-25	99	Northern
4988-25	98	Central
5158-25	100	Central
4786-25	100	Central
4756-25	100	Jerusalem
4935-25	98	Jerusalem
5580-25	100	Central
5599-25	100	Jerusalem
5601-25	100	Central
5604-25	100	Central
5605-25	100	Jerusalem
5612-25	100	Jerusalem
5638-25	100	Central
5635-25	100	Northern
5632-25	100	Northern
5651-25	100	Jerusalem
127-26	100	Tel Aviv
180-26	100	Jerusalem
181-26	100	Jerusalem
199-26	100	Central
241-26	100	Central
242-26	100	Northern
258-26	100	Northern

Sequence ID	Sequencing coverage (%)	District
314-26	100	Northern
39-26	100	Central
60-26	100	Jerusalem
354-26	98	Central
366-26	100	Haifa
381-26	100	Central
386-26	100	Central
391-26	100	Northern
1813-25	100	Jerusalem
3067-25	100	Tel Aviv
494-26	100	Central
568-26	100	Central
611-26	100	Central
760-26	100	Central
762-26	100	Central

Appendix Table 3. Population proportion (%) and vaccine coverage by district, 2025.

District	Population proportion (%)	Vaccine coverage (%)
Central	29	89
Jerusalem	13	78
Northern	15	92
Tel Aviv	16	88
Southern	15	90
Haifa	12	90

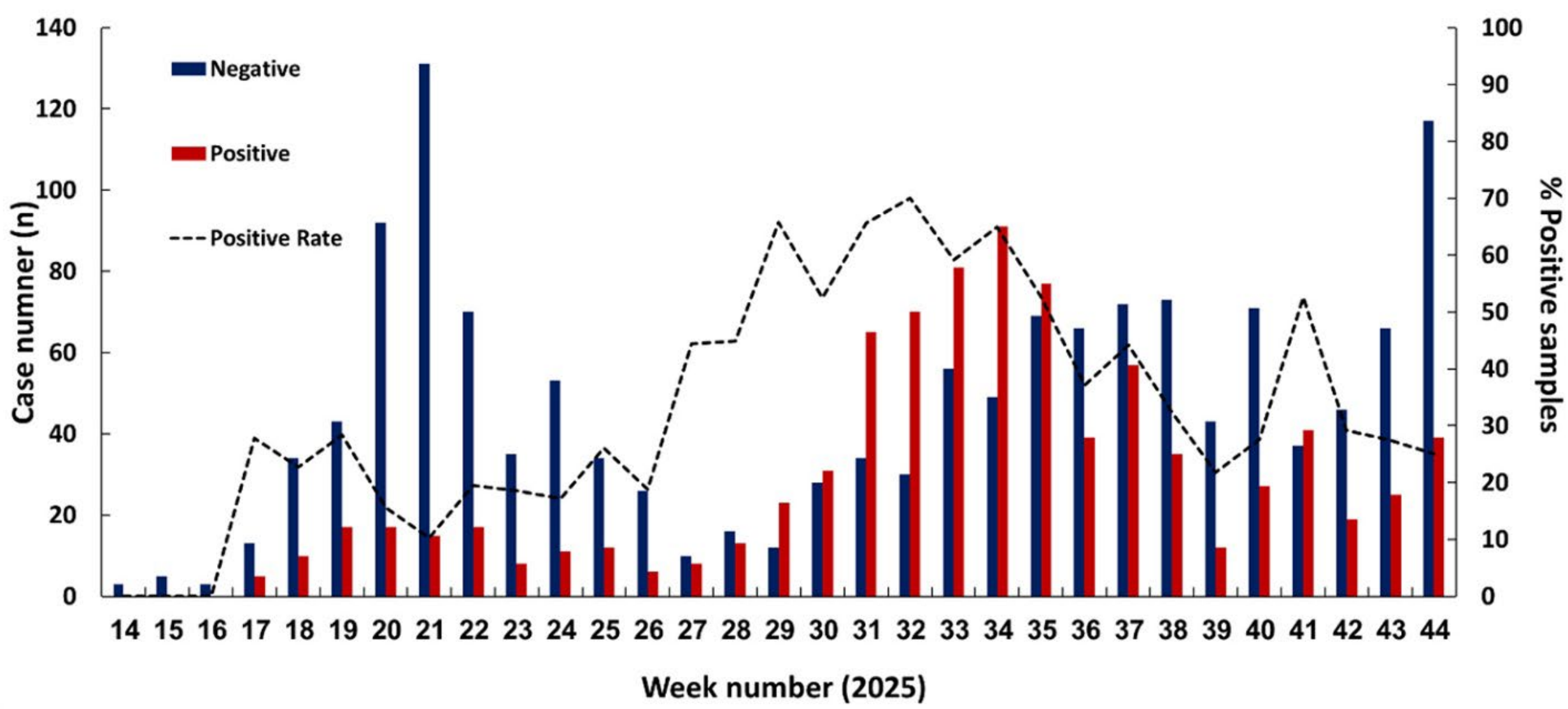

Appendix Figure. Weekly dynamics of molecular laboratory testing. Weekly numbers of measles-positive and negative samples with the corresponding positivity rate. Epidemiological, weeks 14-44 (1 April-31 October 2025).
